# *In vivo *properties of the proangiogenic peptide QK

**DOI:** 10.1186/1479-5876-7-41

**Published:** 2009-06-08

**Authors:** Gaetano Santulli, Michele Ciccarelli, Gianluigi Palumbo, Alfonso Campanile, Gennaro Galasso, Barbara Ziaco, Giovanna Giuseppina Altobelli, Vincenzo Cimini, Federico Piscione, Luca Domenico D'Andrea, Carlo Pedone, Bruno Trimarco, Guido Iaccarino

**Affiliations:** 1 Dipartimento di Medicina Clinica, Scienze Cardiovascolari ed Immunologiche, Cattedra di Medicina Interna, Università degli Studi "Federico II" di Napoli, Italy; 2 Dipartimento di Medicina Clinica, Scienze Cardiovascolari ed Immunologiche, Cattedra di Cardiologia, Università degli Studi "Federico II" di Napoli, Italy; 3 Dipartimento di Scienze Biologiche, Università degli Studi "Federico II" di Napoli, Italy; 4 Dipartimento di Scienze Biomorfologiche e Funzionali, Università degli Studi "Federico II" di Napoli, Italy; 5 Istituto di Biostrutture e Bioimmagini, Consiglio Nazionale delle Ricerche, Napoli, Italy

## Abstract

The main regulator of neovascularization is Vascular Endothelial Growth Factor (VEGF). We recently demonstrated that QK, a de novo engineered VEGF mimicking peptide, shares *in vitro *the same biological properties of VEGF, inducing capillary formation and organization. On these grounds, the aim of this study is to evaluate *in vivo *the effects of this small peptide. Therefore, on Wistar Kyoto rats, we evaluated vasomotor responses to VEGF and QK in common carotid rings. Also, we assessed the effects of QK in three different models of angiogenesis: ischemic hindlimb, wound healing and Matrigel plugs. QK and VEGF present similar endothelium-dependent vasodilatation. Moreover, the ability of QK to induce neovascularization was confirmed us by digital angiographies, dyed beads dilution and histological analysis in the ischemic hindlimb as well as by histology in wounds and Matrigel plugs. Our findings show the proangiogenic properties of QK, suggesting that also *in vivo *this peptide resembles the full VEGF protein. These data open to new fields of investigation on the mechanisms of activation of VEGF receptors, offering clinical implications for treatment of pathophysiological conditions such as chronic ischemia.

## Introduction

Therapeutic vascular growth is a novel rising area for the treatment of ischemic vascular diseases. Limited options for treatment of chronic ischemic diseases, in particular in patients with severe atherosclerosis, have induced to study new therapeutic approaches based on the possibility to increase the development of collateral circulation [1]. This complex process involves both angiogenesis (creation of new capillaries) and arteriogenesis (enlargement and remodeling of pre-existing collaterals) [2]. In detail, the term angiogenesis refers to the sprouting, enlargement, or intussusceptions of new endothelialized channels and is tightly associated to endothelial cells proliferation and migration in response to angiogenic stimuli, in particular hypoxia. Arteriogenesis is, instead, a result of growth and positive remodeling of pre-existing vessels, forming larger conduits and collateral bridges between arterial networks via recruitment of smooth muscle cells. Unlike angiogenesis, this process is linked to shear stress and local activation of endothelium rather than hypoxia [3]. Nevertheless, these two mechanisms interplay during conditions of chronic ischemia and can be modulated by several growth factors, transcription factors and cytokines [3,4].

In particular, the main regulator of neovascularization in adult life is the system of vascular endothelial growth factor (VEGF), that is expressed as several spliced variants. Among its several isoforms, VEGF_165 _is the one that until now has shown the ability to regulate mechanisms of neovascularization both *in vitro *and *in vivo*. The two main VEGF receptors are VEGFR-1 or fms-like tyrosine kinase 1 (Flt-1) and VEGFR-2 or fetal liver kinase 1 (Flk-1) also known as kinase-insert domain-containing receptor (KDR) [2].

In animal models of chronic ischemia, manoeuvres that increase VEGF levels by intramuscular injection or vascular infusion of adenoviral vectors encoding for VEGF [5,6], or indirectly, for example by physical training or β_2 _adrenergic receptor overexpression in ischemic hindlimb (HL), have shown to improve collateral flow [3,5-7]. In spite of all, clinical trials using gene or protein therapy with VEGF isoforms for treatment of myocardial or peripheral ischemia have been somewhat disappointing indicating the needs to develop new approaches in this field [1,8].

We recently demonstrated that a *de novo *synthesized VEGF mimetic, named QK, shares the same biological properties of VEGF and shows the ability to induce capillary formation and organization *in *vitro [9], and showed to be active in gastric ulcer healing in rodents when administered either orally or systemically [10]. This mimetic is a 15 amino acid peptide which adopts a very stable helical conformation in aqueous solution [11] that resembles the 17–25 α-helical region of VEGF_165_, and binds both VEGFR-1 and 2.

The main purpose of this study is to evaluate *in vivo *the effects of this *de novo *engineered VEGF mimicking peptide on neovascularization, in normotensive Wistar Kyoto (WKY) rats. Therefore, we first assessed the properties of QK performing *ex vivo *experiments of vascular reactivity in WKY common carotid rings [12], and then we evaluated *in vivo *the role of this small peptide studying the angiogenic models of ischemic HL, wound healing and Matrigel plugs.

## Methods

### Peptides

The VEGF mimetic, referred to as QK, is a pentadecapeptide (KLTWQELYQLKYKGI) previously described [9]. We also assessed the effects of a peptide without biological activity and so used as control, VEGF_15 _(KVKFMDVYQRSYCHP) [11], corresponding to the unmodified 14–28 region of VEGF_165_, that remains unstructured and does not bind to VEGFRs, indicating that the helical structure is necessary for the biological activity. The N-terminus of these peptides is capped with an acetyl group, while the C-terminus ends in an amide group. Both peptides were synthesized as previously described [9].

### Animal studies

All animal procedures were performed on 12-week-old (weight 280 ± 19 g) normotensive WKY male rats (Charles River Laboratories, Milan, Italy; n = 66). The animals were coded so that analysis was performed without any knowledge of which treatment each animal had received. Rats were cared for in accordance with the *Guide for the Care and Use of Laboratory Animals *published by the National Institutes of Health in the United States (NIH Publication No. 85-23, revised 1996) and approved by the Ethics Committee for the Use of Animals in Research of "Federico II" University.

### Vascular Reactivity Determined on Common Carotid Rings

After isolation from WKY rats (n = 12), common carotids were suspended in isolated tissue baths filled with 25 mL Krebs-Henseleit solution (in mMol/L: NaCl 118.3, KCl 4.7, CaCl_2 _2.5, MgSO_4 _1.2, KH_2_PO_4 _1.2, NaHCO_3 _25, and glucose 5.6) continuously bubbled with a mixture of 5% CO_2 _and 95% O_2 _(pH 7.38 to 7.42) at 37°C as previously described [13,14]. Endothelium-dependent vasorelaxation was assessed in vessels preconstricted with phenylephrine (10^-6 ^Mol/L) in response to VEGF_15_, VEGF_165_, or QK (10^-8 ^to 10^-6 ^Mol/L), prepared daily. The concentration is reported as the final molar concentration in the organ bath. Endothelium-independent vasorelaxation was tested after mechanical endothelium removal of the endothelial layer.

### Surgical Induction of Hindlimb Ischemia

Animals (n = 21) were anesthetized with tiletamine (50 mg/kg) and zolazepam (50 mg/kg); the right common femoral artery was isolated [3,15] and permanently closed with a non re-absorbable suture while the femoral vein was clamped; through an incision on the artery made distal to the suture, with a plastic cannula connected to an osmotic pump (Alzet 2002, Alza Corporation, Palo Alto, California, USA) placed in peritoneum, we performed a chronic (14 days) intrafemoral artery infusion (10^-7 ^Mol/L) of VEGF_15 _(n = 6), VEGF_165 _(n = 7), or QK (n = 8).

### Digital Angiographies and Collateral Blood Flow Determination

Rats were anaesthetized as described above and the left common carotid exposed as previously described [3]. A flame stretched PE50 catheter was advanced into the abdominal aorta right before the iliac bifurcation, under fluoroscopic visualization (Advantix LCX, General Electrics, Milwaukee, Wisconsin, USA). An electronic regulated injector (ACIST Medical Systems Eden Prairie, Minnesota, USA) was used to deliver with constant pressure (900 psi) 0.2 ml of contrast medium (Iomeron 400, Bracco Diagnostics, Milan, Italy). The cineframe number for TIMI frame count (TFC) assessment was measured with a digital frame counter on the suitable cine-viewer monitor as previously described [15-17]. After angiography, we injected into descending aorta 10^5 ^orange dyed microbeads (15 μm diameter, Triton Technologies, San Diego, California, USA) diluted in 1 ml NaCl 0.9% and then animals were euthanized [16]. *Tibialis anterior *muscles of ischemic HL were collected, fixed by immersion in phosphate buffered saline (PBS, 0.01 M, pH 7.2–7.4)/formalin and then embedded in paraffin to be processed for immunohistology. *Gastrocnemious *samples of the ischemic and non-ischemic HL were collected and frozen with liquid nitrogen and then were homogenized and digested; the microspheres were collected and suspended in *N,N*-dimethylthioformamide. The release of dye was assessed by light absorption at 450 nm [7,16]. Data are expressed as ischemic to non-ischemic muscle ratio.

### Animal Wound Healing

The animals (n = 22) were anesthetized as above and the dorsum was shaved by applying a depilatory creme (Veet, Reckitt-Benckiser, Milano, Italy) and disinfected with povidone iodine scrub. A 20 mm diameter open wound was excised through the entire thickness of the skin, including the *panniculus carnosus *layer [15]. Pluronic gel (30%) containing (10^-6 ^M) VEGF_15 _(n = 6), VEGF_165 _(n = 8), or QK (n = 8) was placed directly onto open wounds, then covered with a sterile dressing. An operator blinded to the identity of the sample measured wound areas every day, for 8 days. Direct measurements of wound region were determined by digital planimetry (pixel area), and subsequent analysis was performed using a computer-assisted image analyzer (ImageJ software, version 1.41, National Institutes of Health, Bethesda, MD, USA). Wound healing was quantified as a percentage of the original injury size.

### Matrigel Plugs

Rats (n = 11), anesthetized as described above, were injected subcutaneously midway on the right and left dorsal sides, using sterile conditions, with 0.8 ml of Matrigel^® ^(BD Biosciences, Bedford, MA, USA), mixed with 16 U heparin and either 10^-6^M VEGF_15 _(n = 3), VEGF_165 _(n = 4), or QK (n = 4). After seven days, the animals were euthanized and the implants were isolated along with adjacent skin to be fixed in 10% neutral-buffered formalin solution and then embedded in paraffin. All tissues were cut in 5 μm sections and slides were counterstained with a standard mixture of hematoxylin and eosin [4]. Quantitative analysis was done by counting the total number of endothelial cells, identified by lectin staining (see immunohistology), in the Matrigel plug in each of 20 randomly chosen cross-sections per each group, at ×40 magnification, using digitized representative high resolution photographic images, with a dedicated software (Image Pro Plus; Media Cybernetics, Bethesda, Maryland, USA).

### Immunohistology

After re-hydration, sections were incubated with *Griffonia (Bandeiraea) simplicifolia I *(GBS-I) biotinylated lectin (Sigma, St. Louis, Missouri, USA) overnight (1:50). GBS-I specific adhesion to capillary endothelium was revealed by a secondary incubation for 1 hour at room temperature with (1:400) horseradish peroxidase conjugated streptavidin (Dako, Glostrup, Denmark), which in presence of hydrogen peroxide and diaminobenzidine gives a brown reaction product. Five tissue sections of each animal from each experimental group were examined. The number of capillaries per 20 fields was measured on each section by two independent operators, blind to treatment [3,15,16]. The differences between groups were evaluated by analysis of variance (ANOVA).

### Statistical Analysis

All data are presented as the mean value ± SEM. Statistical differences were determined by one-way or two-way ANOVA and Bonferroni post hoc testing was performed where applicable. A p value less than 0.05 was considered to be significant. All the statistical analysis and the evaluation of data were performed using GraphPad Prism version 5.01 (GraphPad Software, San Diego, California, USA).

## Results

**Figure 1:**
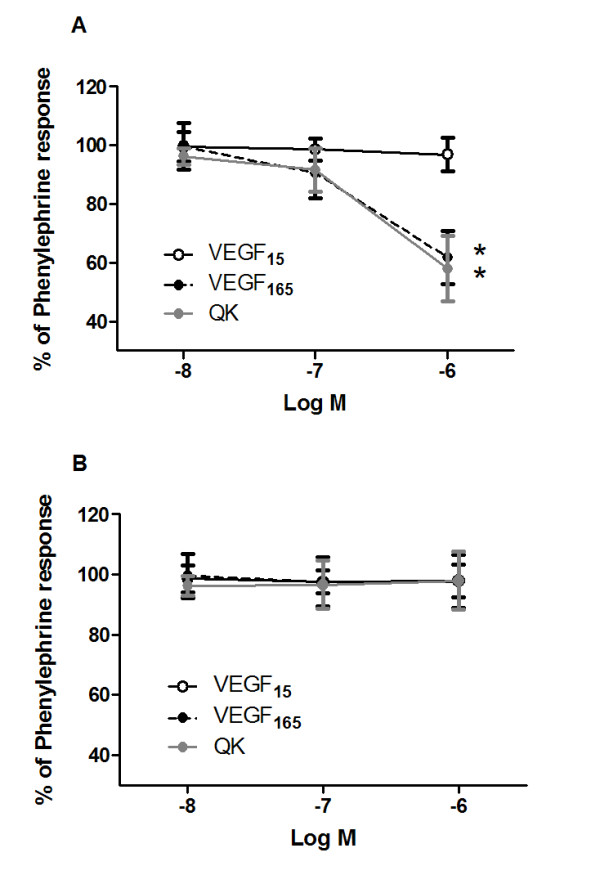
**Effects of VEGF_15_, VEGF_165 _and QK on the vasomotor responses of 12 common carotid arteries from normotensive rats (A)**. Both VEGF_165 _and QK induced a comparable vasorelaxation, while VEGF_15_, has no evident effect. After removal of the endothelial layer there is no appreciable vasorelaxation (**B**). * = p < 0.05 vs VEGF_15_. Error bars show SEM.

**Figure 2:**
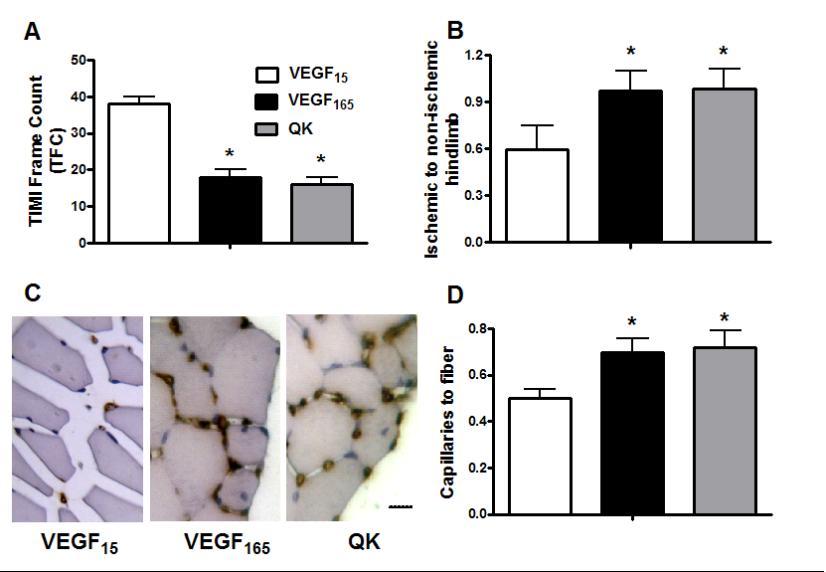
**In the model of ischemic hindlimb, VEGF_165 _as well QK enhanced and ameliorated regenerative responses, as assessed by TIMI Frame Count (TFC, Panel A), dyed beads dilution from *gastrocnemious *muscles (B) and of histological analysis, with representative images (C) of lectin GBS-I staining of capillaries in the *tibialis anterior *muscle**. (Magnification ×40; bar = 10 μm) and the evaluation as number of capillaries per number of fibers (**D**) * = p < 0.05 vs VEGF_15_. Error bars show SEM.

**Figure 3:**
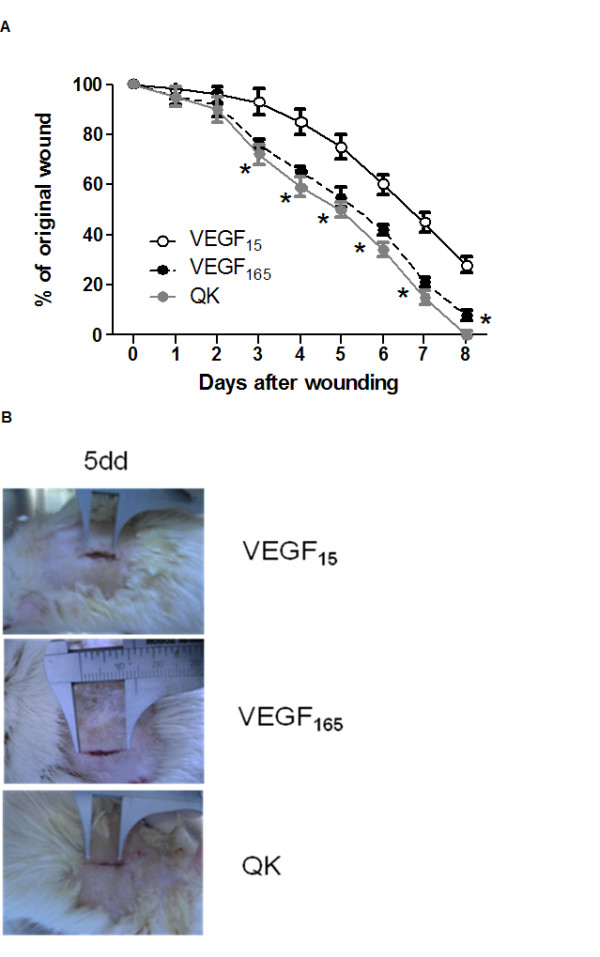
**Diagram of the kinetics of wound closure (A)**. VEGF_165 _and QK accelerate the closure of full thickness punch biopsy wounds. Three to five rats were analyzed at each time point. Gross appearance after 5 days of the wound treated with VEGF_15_, VEGF_165_, QK (10^-6^M); * = p < 0.05 vs VEGF_15_. Representative digital photographs (**B**) 5 days after wound. Error bars show SEM.

**Figure 4:**
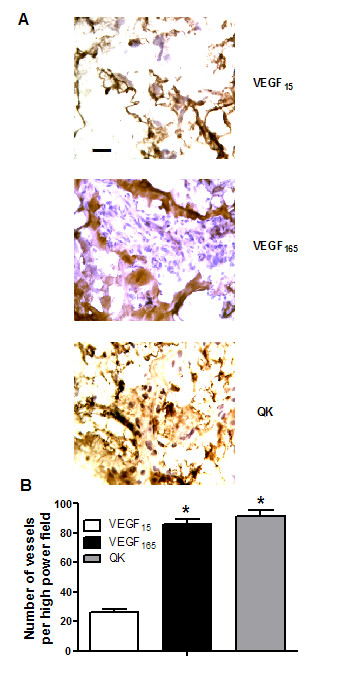
**Representative images of Matrigel plugs subcutaneously injected at a magnification of ×60; bar = 40 μm**. Endothelial cells are identified by lectin staining, that gives a brown reaction product. Different background is due to counterstaining, performed with a standard mixture of hematoxylin and eosin, as described in Methods (**A**). Quantification of microvessels infiltrating Matrigel plugs (**B**). * = p < 0.05 vs VEGF_15_. Error bars show SEM.

Properties of QK were first assessed in *ex vivo *experiments of vascular reactivity (Figure 1), and then in three different *in vivo *regenerative models (Figures 2, 3 and 4), so to show the ability of QK to induce neovascularization.

### Vascular reactivity

Vasomotor responses showed a similar relaxation induced by 10^-6 ^M VEGF_165 _and QK while, as expected, substantially no action was detected after VEGF_15 _administration. (Figure 1A). The endothelium was mechanically removed from the aortic rings to assess endothelium-independent vasomotor responses. Gentle endothelium denudation prevented QK and VEGF_165 _vasorelaxation, indicating that these responses are endothelium dependent (Figure 1B).

### Ischemic hindlimb

Ischemic HL perfusion was assessed by TFC score of digital microangiographies. Both VEGF_165 _and QK ameliorated the TFC score (VEGF_165_:17 ± 2; QK:16 ± 2) compared to the scramble peptide-infused HL (VEGF_15_:38 ± 3; p < 0.05, ANOVA) as depicted in Figure 2A.

Regional *gastrocnemius *blood flow was also measured by dyed microspheres entrapment after intra-aortic infusion. After muscle digestion, dye elution is properly related to HL perfusion (ischemic/not-ischemic) [3]. Once again (Figure 2B), VEGF_165 _and QK treatment achieved a better ischemic HL perfusion than VEGF_15 _treatment (VEGF_165_:0.92 ± 0.1; QK:0.95 ± 0.1; VEGF_15_:0.59 ± 0.2; p < 0.05, ANOVA).

Capillary density was assessed on the *tibialis anterior *muscle of the ischemic HL by means of lectin istochemistry. VEGF_165 _and QK increased capillaries to muscle fibers ratio in comparison with VEGF_15 _(VEGF_15_:0.5 ± 0.04; VEGF_165_:0.7 ± 0.06; QK:0.72 ± 0.07; p < 0.05, ANOVA), as shown in Figure 2C, D.

### Wound healing

The examination of full-thickness wounds in the back skin shows that both QK and VEGF_165 _accelerate healing by enhancing angiogenesis in the granulation tissue (Figure 3).

### Matrigel plugs

After injection, Matrigel containing the angiogenic stimuli forms a plug into which blood vessels can migrate. Matrigel pellets evidenced a significant greater peripheral capillaries infiltration in VEGF_165 _(86 ± 3.0) and QK (91 ± 4.5) treated rats than in VEGF_15 _ones (26 ± 2.0; p < 0.05 vs VEGF_165 _and QK, ANOVA), as shown in Figure 4.

## Discussion

In the present study, we examinated the *in vivo *effects of a VEGF_165 _mimetic, named QK, modeled on the region of the VEGF protein responsible for binding to and activating the VEGFRs that are known to trigger angiogenesis. We previously showed that QK can bind to the VEGFRs, initiate VEGF-induced signaling cascades and stimulate angiogenesis *in vitro *[9]. This is the first report to show that this peptide is able to recapitulate the *in vivo *responses of VEGF.

Angiogenesis is known to be a process of new blood vessel formation from a pre-existing endothelial structure. It is tuned by proangiogenic and antiangiogenic factors, and the shift from this equilibrium may lead to pathological angiogenesis [18,19]. Indeed, deregulation of angiogenesis is involved in several conditions including cancer, ischemic, and inflammatory diseases (atherosclerosis, rheumatoid arthritis, or age-related macular degeneration). Therefore, the research for drugs able to regulate angiogenesis constitutes a pivotal research field. In particular, occlusive vascular disease remains an important cause for death and morbidity in industrialized society [1,20], despite efforts to design new and efficient treatment strategies [19,21].

Unfortunately, numerous reports indicate that in laboratory animals over-expression of VEGF may lead to metabolic dysfunction, formation of leaky vessels and transient edema [1,22]. Indeed, VEGF actions include the induction of endothelial cells proliferation and migration; it is also known as a vascular permeability factor, based on its ability to induce vascular leakage and vasodilatation in a dose dependent fashion as a result of endothelial cell-derived nitric oxide [12,23].

In humans, various clinical trials were designed to verify new vessel growth by exogenous administration of proangiogenic factors in patients with refractory ischemic symptoms. Albeit initial small open-labeled trials yielded promising results, subsequent larger double-blind randomized placebo-controlled clinical trials have failed to show much clinical benefit [19,24,25]. These largely disappointing results may in part be explained by suboptimal delivery of genetic material to target cells or tissue. Moreover, although adenoviral vectors provide high levels of gene transfer and expression, there are well known virus-related adverse effects, such as the induction of immune and inflammatory response [6,21,26]. Recently, several side effects have been reported for VEGF administration in human subjects [1,8,25] such as increase in atherosclerotic plaques, lymphatic edema or uncontrolled neoangiogenesis leading to the development of functionally abnormal blood vessels, so to preclude its use in a large share of ischemic population [21,27].

A hopeful alternative could be to use angiogenic stimulators of smaller size, such as peptides, with a well-characterized biologic mechanism of action. Indeed, recent reports revealed a specific antagonistic relationship between VEGF and other vascular growth factors, such as the placental growth factor (PlGF), the basic fibroblast growth factor (bFGF) and the platelet-derived growth factor (PDGF), with a dichotomous role for VEGF and VEGFRs [28-30]. So, the function of VEGF is far more intricate: it can also negatively regulate angiogenesis and tumorigenesis, by impeding the function of the PDGF receptor on pericytes, leading to a loss of pericyte coverage of blood vessels [31]. Moreover, several studies demonstrated a more efficacious action obtained with a specific stimulation of VEGFRs [32,33] if compared to VEGF overexpression [22,34]. These findings suggest that the multifaceted array of the biological responses linked to VEGF may be ascribable to its proneness to dimerize or interact with other molecules [29]. Thus, because of lower molecular and biological complexity, peptides that ensure only the needed interaction with specific receptors could be candidate lead compounds for a safer proangiogenic drug, also to avoid adverse effects.

### Perspectives

We show that the VEGF mimetic QK is able to increase neoangiogenesis and collateral flow in WKY rats. Our findings evidence the proangiogenic properties of this small peptide, suggesting that also *in vivo *QK resembles the full VEGF protein. Thus, a single peptide, that would not be expected to dimerize, is still able to induce VEGF specific angiogenic responses. Clearly, further studies are needed to fully understand this mechanism, that appears of intriguing interest. Anyway, these data open to new fields of investigation on the mechanisms of activation of VEGFRs, also to clarify complex angiogenesis pathways, with strong clinical implications for treatment of pathophysiological conditions such as chronic ischemia.

## Competing interests

The authors declare that they have no competing interests.

## Authors' contributions

GS, GI, MC, LDDA, CP and BT designed research, GS, MC, GP, AC, GG, BZ, GGA, VC, and FP, carried out the experiments; GS and GI performed the statistical analysis; GS, GI and BT drafted the manuscript. All authors read and approved the final manuscript.

## References

[B1] Schaper W (2009). Collateral circulation: past and present. Basic Res Cardiol.

[B2] Testa U, Pannitteri G, Condorelli GL (2008). Vascular endothelial growth factors in cardiovascular medicine. J Cardiovasc Med (Hagerstown).

[B3] Iaccarino G, Ciccarelli M, Sorriento D, Galasso G, Campanile A, Santulli G, Cipolletta E, Cerullo V, Cimini V, Altobelli GG, Piscione F, Priante O, Pastore L, Chiariello M, Salvatore F, Koch WJ, Trimarco B (2005). Ischemic neoangiogenesis enhanced by beta2-adrenergic receptor overexpression: a novel role for the endothelial adrenergic system. Circ Res.

[B4] Li J, Post M, Volk R, Gao Y, Li M, Metais C, Sato K, Tsai J, Aird W, Rosenberg RD, Hampton TG, Sellke F, Carmeliet P, Simons M (2000). PR39, a peptide regulator of angiogenesis. Nat Med.

[B5] Takeshita S, Zheng LP, Brogi E, Kearney M, Pu LQ, Bunting S, Ferrara N, Symes JF, Isner JM (1994). Therapeutic angiogenesis. A single intraarterial bolus of vascular endothelial growth factor augments revascularization in a rabbit ischemic hind limb model. J Clin Invest.

[B6] Vajanto I, Rissanen TT, Rutanen J, Hiltunen MO, Tuomisto TT, Arve K, Narvanen O, Manninen H, Rasanen H, Hippelainen M, Alhava E, Ylä-Herttuala S (2002). Evaluation of angiogenesis and side effects in ischemic rabbit hindlimbs after intramuscular injection of adenoviral vectors encoding VEGF and LacZ. J Gene Med.

[B7] Leosco D, Rengo G, Iaccarino G, Filippelli A, Lymperopoulos A, Zincarelli C, Fortunato F, Golino L, Marchese M, Esposito G, Rapacciuolo A, Rinaldi B, Ferrara N, Koch WJ, Rengo F (2007). Exercise training and beta-blocker treatment ameliorate age-dependent impairment of beta-adrenergic receptor signaling and enhance cardiac responsiveness to adrenergic stimulation. Am J Physiol Heart Circ Physiol.

[B8] Lei Y, Haider H, Shujia J, Sim ES (2004). Therapeutic angiogenesis. Devising new strategies based on past experiences. Basic Res Cardiol.

[B9] D'Andrea LD, Iaccarino G, Fattorusso R, Sorriento D, Carannante C, Capasso D, Trimarco B, Pedone C (2005). Targeting angiogenesis: structural characterization and biological properties of a de novo engineered VEGF mimicking peptide. Proc Natl Acad Sci USA.

[B10] Dudar GK, D'Andrea LD, Di Stasi R, Pedone C, Wallace JL (2008). A vascular endothelial growth factor mimetic accelerates gastric ulcer healing in an iNOS-dependent manner. Am J Physiol Gastrointest Liver Physiol.

[B11] Diana D, Ziaco B, Colombo G, Scarabelli G, Romanelli A, Pedone C, Fattorusso R, D'Andrea LD (2008). Structural determinants of the unusual helix stability of a de novo engineered vascular endothelial growth factor (VEGF) mimicking peptide. Chemistry.

[B12] Fukumura D, Gohongi T, Kadambi A, Izumi Y, Ang J, Yun CO, Buerk DG, Huang PL, Jain RK (2001). Predominant role of endothelial nitric oxide synthase in vascular endothelial growth factor-induced angiogenesis and vascular permeability. Proc Natl Acad Sci USA.

[B13] Ciccarelli M, Cipolletta E, Santulli G, Campanile A, Pumiglia K, Cervero P, Pastore L, Astone D, Trimarco B, Iaccarino G (2007). Endothelial beta2 adrenergic signaling to AKT: role of Gi and SRC. Cell Signal.

[B14] Iaccarino G, Ciccarelli M, Sorriento D, Cipolletta E, Cerullo V, Iovino GL, Paudice A, Elia A, Santulli G, Campanile A, Arcucci O, Pastore L, Salvatore F, Condorelli G, Trimarco B (2004). AKT participates in endothelial dysfunction in hypertension. Circulation.

[B15] Sorriento D, Ciccarelli M, Santulli G, Campanile A, Altobelli GG, Cimini V, Galasso G, Astone D, Piscione F, Pastore L, Trimarco B, Iaccarino G (2008). The G-protein-coupled receptor kinase 5 inhibits NFkappaB transcriptional activity by inducing nuclear accumulation of IkappaB alpha. Proc Natl Acad Sci USA.

[B16] Ciccarelli M, Santulli G, Campanile A, Galasso G, Cervero P, Altobelli GG, Cimini V, Pastore L, Piscione F, Trimarco B, Iaccarino G (2008). Endothelial alpha1-adrenoceptors regulate neo-angiogenesis. Br J Pharmacol.

[B17] Galasso G, Schiekofer S, Sato K, Shibata R, Handy DE, Ouchi N, Leopold JA, Loscalzo J, Walsh K (2006). Impaired angiogenesis in glutathione peroxidase-1-deficient mice is associated with endothelial progenitor cell dysfunction. Circ Res.

[B18] Carmeliet P (2000). VEGF gene therapy: stimulating angiogenesis or angioma-genesis?. Nat Med.

[B19] Khurana R, Simons M, Martin JF, Zachary IC (2005). Role of angiogenesis in cardiovascular disease: a critical appraisal. Circulation.

[B20] Sirico G, Brevetti G, Lanero S, Laurenzano E, Luciano R, Chiariello M (2009). Echolucent femoral plaques entail higher risk of echolucent carotid plaques and a more severe inflammatory profile in peripheral arterial disease. J Vasc Surg.

[B21] Epstein SE, Kornowski R, Fuchs S, Dvorak HF (2001). Angiogenesis therapy: amidst the hype, the neglected potential for serious side effects. Circulation.

[B22] Karpanen T, Bry M, Ollila HM, Seppanen-Laakso T, Liimatta E, Leskinen H, Kivela R, Helkamaa T, Merentie M, Jeltsch M, Paavonen K, Andersson LC, Mervaala E, Hassinen IE, Ylä-Herttuala S, Oresic M, Alitalo K (2008). Overexpression of vascular endothelial growth factor-B in mouse heart alters cardiac lipid metabolism and induces myocardial hypertrophy. Circ Res.

[B23] Gigante B, Morlino G, Gentile MT, Persico MG, De Falco S (2006). Plgf-/-eNos-/- mice show defective angiogenesis associated with increased oxidative stress in response to tissue ischemia. FASEB J.

[B24] Henry TD, Annex BH, McKendall GR, Azrin MA, Lopez JJ, Giordano FJ, Shah PK, Willerson JT, Benza RL, Berman DS, Gibson CM, Bajamonde A, Rundle AC, Fine J, McCluskey ER, VIVA Investigators (2003). The VIVA trial: Vascular endothelial growth factor in Ischemia for Vascular Angiogenesis. Circulation.

[B25] Isner JM, Vale PR, Symes JF, Losordo DW (2001). Assessment of risks associated with cardiovascular gene therapy in human subjects. Circ Res.

[B26] Brevetti LS, Sarkar R, Chang DS, Ma M, Paek R, Messina LM (2001). Administration of adenoviral vectors induces gangrene in acutely ischemic rat hindlimbs: role of capsid protein-induced inflammation. J Vasc Surg.

[B27] Celletti FL, Waugh JM, Amabile PG, Brendolan A, Hilfiker PR, Dake MD (2001). Vascular endothelial growth factor enhances atherosclerotic plaque progression. Nat Med.

[B28] Cao Y, Linden P, Shima D, Browne F, Folkman J (1996). In vivo angiogenic activity and hypoxia induction of heterodimers of placenta growth factor/vascular endothelial growth factor. J Clin Invest.

[B29] Eriksson A, Cao R, Pawliuk R, Berg SM, Tsang M, Zhou D, Fleet C, Tritsaris K, Dissing S, Leboulch P, Cao Y (2002). Placenta growth factor-1 antagonizes VEGF-induced angiogenesis and tumor growth by the formation of functionally inactive PlGF-1/VEGF heterodimers. Cancer Cell.

[B30] Greenberg JI, Shields DJ, Barillas SG, Acevedo LM, Murphy E, Huang J, Scheppke L, Stockmann C, Johnson RS, Angle N, Cheresh DA (2008). A role for VEGF as a negative regulator of pericyte function and vessel maturation. Nature.

[B31] Stockmann C, Doedens A, Weidemann A, Zhang N, Takeda N, Greenberg JI, Cheresh DA, Johnson RS (2008). Deletion of vascular endothelial growth factor in myeloid cells accelerates tumorigenesis. Nature.

[B32] Smadja DM, Bieche I, Helley D, Laurendeau I, Simonin G, Muller L, Aiach M, Gaussem P (2007). Increased VEGFR2 expression during human late endothelial progenitor cells expansion enhances in vitro angiogenesis with up-regulation of integrin alpha(6). J Cell Mol Med.

[B33] Wang D, Donner DB, Warren RS (2000). Homeostatic modulation of cell surface KDR and Flt1 expression and expression of the vascular endothelial cell growth factor (VEGF) receptor mRNAs by VEGF. J Biol Chem.

[B34] Masaki I, Yonemitsu Y, Yamashita A, Sata S, Tanii M, Komori K, Nakagawa K, Hou X, Nagai Y, Hasegawa M, Sugimachi K, Sueishi K (2002). Angiogenic gene therapy for experimental critical limb ischemia: acceleration of limb loss by overexpression of vascular endothelial growth factor 165 but not of fibroblast growth factor-2. Circ Res.

